# First person – Manuela Santalla

**DOI:** 10.1242/bio.058569

**Published:** 2021-02-15

## Abstract

First Person is a series of interviews with the first authors of a selection of papers published in Biology Open, helping early-career researchers promote themselves alongside their papers. Manuela Santalla is first author on ‘Testing the effect of tobacco cigarettes on heart function of *Drosophila melanogaster*’, published in BiO. Manuela is a postdoc in the lab of Dr. Paola Ferrero, interested in understanding how the heart works. She studies the physiology of the heart of the fruit fly and how different conditions affect it.


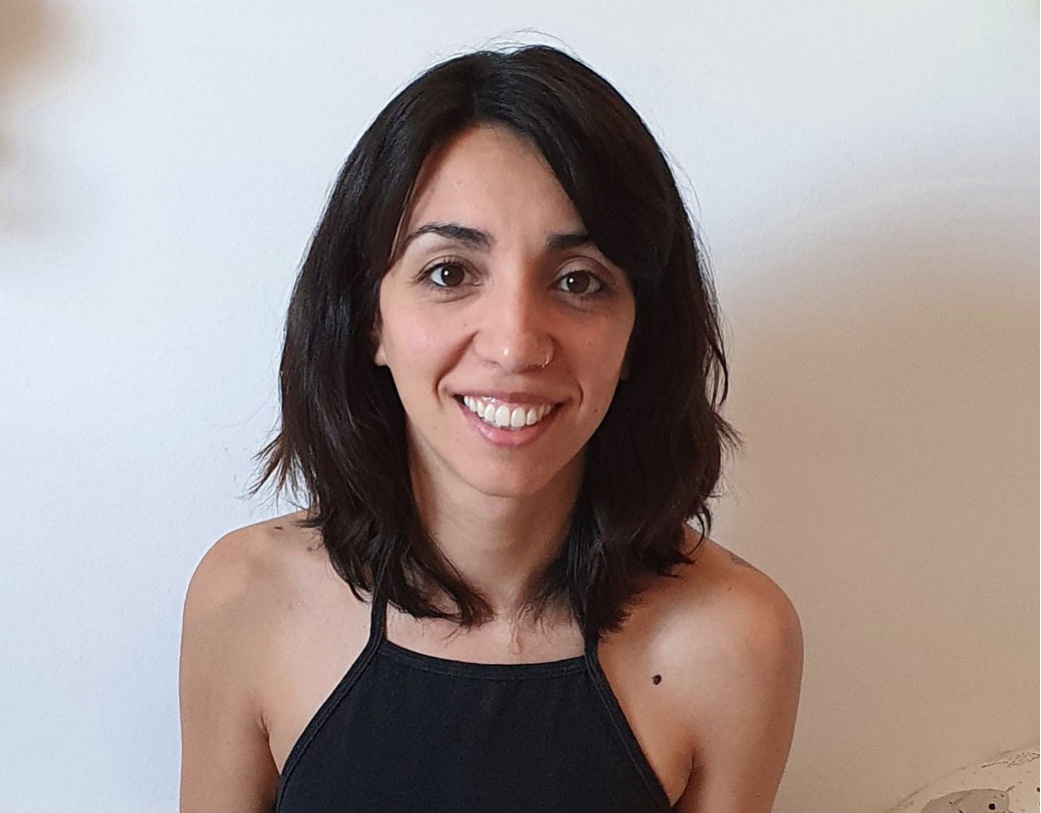


**Manuela Santalla**

**What is your scientific background and the general focus of your lab?**

I have a degree in genetics from the National University of the Northwest of Buenos Aires and a PhD in biological sciences from the National University of La Plata, Argentina. During my PhD studies, I focused on the effects of differential expression of two proteins involved in translation in parallel to studies about the effect of aging and the consumption of some substances like caffeine and cannabinoids on the heart performance in *Drosophila*.

We are interested in learning about different cardiac physiopathological processes. In this context, as a postdoctoral scholar, I'm studying an endemic disease in Latin America, Chagas disease, because its main consequence is Chagasic cardiomyopathy.

“I'm studying an endemic disease in Latin America, Chagas disease …”

**How would you explain the main findings of your paper to non-scientific family and friends?**

In this work, we demonstrate the effect of tobacco smoke and nicotine on the heart function of the fruit fly mediating a customized device.

Tobacco is one of the substances most consumed by the world population. Its effects at the respiratory level are well known, but how it affects other organs and tissues has been less studied. We studied the effects of tobacco smoke on the heart, using the fruit fly, *Drosophila melanogaster* due to the feasibility of this model to reproduce aspects of human diseases. Using this organism as a model, we can explore the relevance of conserved genes in several diseases. We found that exposure to tobacco smoke negatively affects the fly's heart and the nicotine is largely responsible for the effects we found. This provides an additional understanding of the action of neonicotinoids, used as insecticides, on insect physiology. We found that two genes that codify for alpha 1 and alpha 7 subunits of the nicotine receptor are partially responsible for these effects.

**What are the potential implications of these results for your field of research?**

We have demonstrated the effects of tobacco smoke on the heart of *Drosophila*. This has two main implications, on the one hand, results contribute to understanding how the consumption of this substance affects the heart performance in humans, validating some discovered consequences of tobacco in chronic smokers and exploring the relevance of genes that compounds the nicotinic receptors. On the other hand, our results contribute to the understanding of the mechanism of action of neonicotinoids used as insecticides, beyond the knowledge effects on the central nervous system. Approaches destined to know the mechanisms by which tobacco compounds act might be useful to understand the cardiac consequences of tobacco use and abuse. In addition, the advantage of using the fruit fly as a model is the independence between the cardiac and respiratory systems. This, allows us to study the effects of tobacco smoke directly on the heart, dissociated from the effects at the lung level that smoke produces in humans.

“…the advantage of using the fruit fly as a model is the independence between the cardiac and respiratory systems.”

**What has surprised you the most while conducting your research?**

I think the most interesting issue about research is the possibility of it surprising you every day. That is why, although we build our hypotheses and expect certain results, I am always open to ‘what will happen?’

When we started this research, I didn't know what to expect. Thus, finding that *Drosophila* hearts were affected by tobacco smoke was fascinating. The influence of nicotine as the cause of effects on heart activity and the relevance of the genetic constitution in the interaction with substances and the environment seems amazing. It is really exciting to be able to understand how biology works. Moreover, given the genetic similarity between the fruit fly and humans, understanding that the same phenomena might happen in our hearts seems magnificent to me.

**What, in your opinion, are some of the greatest achievements in your field and how has this influenced your research?**

Despite the evolutive distance, the use of *Drosophila* as a model for understanding biological mechanisms in mammals and especially in humans is surprising. Over the past few years, we have seen published advances on *Drosophila* heart performance as findings like the Bowditch effect – described in mammals – and effects of several compounds like caffeine, nicotine, and cannabinoids on heart performance. At present, we afford genetic models of diseases like Parkinsonism and epilepsy.

**What changes do you think could improve the professional lives of early-career scientists?**

I think that considering early-career scientists as workers and not just cheap labor is a great start. In general, early-career scientists work hard for little money. Our work needs to be valued. In Argentina we are lucky to be able to make a career in science. The State is in charge of financing science and research but, unfortunately, economic crises affect us hard. We are never a priority. I believe that more investment in science and education is fundamental for the growth of any country, improving not only the quality of life of researchers but also of all citizens. But, as for early-career researchers, having a better investment in science would allow us to improve our work in and for our country. Finally, lack of resources makes us creative, without missing the scientific rigor to be competitive at the international level.

**What's next for you?**

I'd really like to keep researching. This year I am finishing my postdoc and we are considering a move to do a postdoc in Europe with my family (I have an 18-month-old baby). But, nonetheless, it all depends on the sanitary conditions. Later, I would like to settle in Argentina and research here, in the universities that trained me.
